# Parting Gold and Silver on the Large Scale: Being Notes of a Visit to the Chemical Department of the United States Mint in Philadelphia

**Published:** 1854-01

**Authors:** A. Snowden Piggot


### ARTICLE IV.

*Parting Gold and Silver on the Large Scale: being Notes of a Visit to the Chemical Department of the United States Mint in Philadelphia.* Read before the Baltimore Academy of Science and Art. By A. SNOWDEN PIGGOT, M. D.

As far as I know, the United States Mint, in Philadelphia, contains the largest establishment in the world for refining the precious metals. A visit to this institution, therefore, is of peculiar interest to the chemist and metallurgist, as he there has an opportunity of witnessing the effects of quantity in influencing the chemical affinities which, from the subject of his daily study, as well as of observing the application of the principles discovered in the laboratory to the great manufacturing processes of the arts. I have, therefore, concluded to give a brief account of the operation of parting, as there conducted, for the opportunity of examining which, I am indebted to the politeness of Prof. James C. Booth.

The chemical department of the Mint consists of an extensive range of rooms in several stories. The distribution of these rooms being a mere matter of convenience, I shall not stop to describe it; neither shall I be guided in my account by any mere architectural arrangement.

The first of the processes is, of course, the fusion of the metals designed to be parted. This is effected in one of Barron's furnaces, of a peculiar, yet simple construction. It consists of a fire-place and two ovens. The fire-place is cylindrical, and is fed from a cylindrical hopper resting upon it, filled with coal. Beneath the fire-place is an air-drum, which receives the nozzles of these blast-pipes. Barron's patent, it will be remembered, consists in a modification of the tuyéres of the blast-furnace. Instead of luting the pipes of the tuyére, so that all the air which enters the furnace must pass through the former, he leaves a small space all around the pipes, between them and the tuyére. This, it is believed, has the effect of greatly increasing the draught by carrying in a large volume of air outside of the pipes, so that this is a combination of the blast and wind furnaces. In the furnace under consideration, two tubes, one on each side, pass from the central cylinder which contains the coal into the lateral ovens. These, of course, contain no fuel, but are heated by the flame and hot gas passing off from the fire. They are cylindrical, and each is closed above like the common metallurgic wind-furnace, with a tile. The crucible (of black lead) is placed in the centre of the oven.

The proportion, in which the metals are alloyed in this preliminary fusion, is two parts of silver to one of gold. The old proportion was three of silver to one of gold, whence the old name, *quartation*, applied to this process. Pettenkofer considers one-and-three-quarters of silver to one of gold sufficient for all practical purposes.

The metals, thus fused and alloyed, are granulated by pouring them, still in a state of fusion, into water which is kept agitated. Much, of course, depends upon the minuteness of the subdivision effected by this operation. The more complete that is, the more readily will the subsequent separation of the metals from one another be accomplished.

The process of parting adopted here is a slight modification of the old Mexican method. The reason assigned by the chemist for preferring the nitric to the sulphuric acid parting, was the position of the Mint in the heart of the city of Philadelphia. The vapors from so large a quantity of sulphuric acid would be intolerable, and the difficulty and expense of preventing their escape would not be paid for by the increased purity of the silver obtained. He says, also, that he is able to work as closely as necessary with nitric acid, and that his *surcharge* is not objectionable, and that his silver contains but little gold.

The granulations are introduced into porcelain pots, and the residual nitric acid of a former operation, presently to be described, is poured upon them. The acid used make about 38° in Baume's hydrometer. There are 60 of these porcelain pots, holding each about 150 pounds of the alloy, so that the amount operated upon at one time, may be 9000 pounds, consisting of

3000 pounds of gold and 6000 pounds of silver. Usually, however, somewhat less than this, say 600,000 dollars worth of the metal are parted at once. These pots are placed in the draught of a tall chimney, in large wooden chambers, closed by doors which rise and fall on a pulley. This whole contrivance is so well managed, that, although such large quantities of acid are used, not the slightest unpleasant odor can be perceived about the premises.

The pots are heated with steam for 5 or 6 hours each day, and the mass is repeatedly stirred in order to present fresh surfaces to the acid, and to break down the lumps of gold which are left, and which necessarily shield some silver from the action of the solvent. After the steam is shut off, the action continues with great violence, generating a very considerable amount of heat, so that the next day the pots are still warm.

This process extracts by far the greater portion of the silver, leaving not more than 1 or 2 per cent. still combined with the gold. The next morning, after the liquid has become quiet, it is drawn off, and a fresh charge of acid poured upon the remaining metal. This extracts the rest of the silver down to a few thousandths, which it is of no consequence to separate. The gold is now taken out and thoroughly washed with steam-heated water on large cloth filters, and the acid is returned to the pots to be used in a new operation. The siphon used for these operations, is quite large, made of gold, and valued at 3000 dollars. It is annually returned as bullion.

The liquid drawn off from the pots is poured into a large wooden vat, holding from 1200 to 1500 gallons, where the chloride of silver is precipitated by means of common salt in solution. The precipitation being complete, the chloride is drawn off into square filters of coarse muslin. Here it is washed unintermittingly by a stream of water from a hose. It is necessary that this stream should continue to flow during the entire washing. Should it be intermitted, the chloride will sink down upon the filter and speedily become so condensed as to be with difficulty penetrated by the water. The washing is kept up until the liquid which passes through is "sweet," as the workmen say, that is, till it is neutral to test paper. It is commonly continued till 1 o'clock, A. M.

The washings from the gold, which contain about one-fourth of all the silver, are treated separately. They are conveyed to a lower floor and there precipitated.

The chloride, thus washed, is transferred to four large vats in which it is decomposed, metallic silver being precipitated. This is effected by granulated zinc which is added in excess, the residual zinc being dissolved by sulphuric acid. No acid is necessary to start the operation, the chloride, in such large quantities, acting with sufficient energy by itself and generating considerable heat. I was surprised to learn that the chloride and sulphate of zinc thus obtained were not preserved, the manufacturers of zinc white not being willing to buy them, and the wholesale apothecaries finding enough in one day's results to stock the market for a long time.

The silver thus obtained is washed to free it from all adhering impurities and transferred to a lower floor. Here it is packed into a drum or short cylinder, and subjected to the action of a very powerful hydraulic press, worked by steam. By this process it is of course greatly condensed, assuming a brilliant lustre in places on the circumference of the cylinder, and much liquid runs out of it. Minute particles of silver flow out with the liquid, and a simple but ingenious trap is contrived to save this valuable sludge. A box with compartments, resembling somewhat an old galvanic battery in form, is placed so as to receive the current of water issuing from the press. Each compartment is perforated about its middle with a horizontal slit, so narrow as to permit the fluid to flow out very gradually. In this manner, every portion of the steam is detained some time in each cell, and the metal contained in it, settles down in the first cells of the series, as an impalpable mud. Over the top of the last cell runs the clear liquid entirely stripped of its silver, which has been deposited in the first cells in which it had been received.

The gold resulting from the parting is in very fine powder, almost as minute as though it had been precipitated by sulphate of iron. It is of a rich sienna-brown approaching to black and leaving here and there a purple stain. It is worked in the same way as the silver by the hydraulic press. The masses coming from this press resemble great cheeses more than any thing else I can compare them to. The gold becomes yellower and the silver whiter, but the latter does not entirely lose its ashen tint except upon the edges where it has been forced against the polished walls of the cylinder.

These cakes, weighing about forty pounds each, are taken up into the melting room, where they are fused and alloyed. For this purpose copper only is used. This gives to the gold pieces a yellow color, which approaches a red instead of that bright brassy yellow which is attained by the mixture of silver and copper in the coin. The difference will be very perceptible, if a gold dollar is compared with an old half eagle.

It will thus be perceived that the operations in the Mint differ little, chemically, from the old process of nitric acid parting. The chief improvements which have been made by the present chemist are the enlargement of the apparatus, to meet the increased demands made upon the Mint by California, and the increase of the facilities for transporting the products of the different operations to the point at which they are needed. All the vessels which contain these, move upon wheels, and arrangements are made for transferring not only the solids, but the liquids from one story to another with ease and rapidity. Hose are provided to supply a constant stream of water for washing.

The closeness with which the metals are worked, may be learned from the annual reports to the Treasury. Much of the burden in the way of parting, &c., now thrown upon the Mint, will, however, probably be taken off by the recently established Assay Office in New York. If that should be well conducted, it may do all the refining of bullion, &c., for private individuals, leaving to the Mint only its peculiar duty of manufacturing the coin of the country and performing the chemical operations therewith connected.

